# Structured tools for assessing quality and risk of bias in Mendelian randomization studies: an updated systematic review

**DOI:** 10.1093/ije/dyag052

**Published:** 2026-04-18

**Authors:** Jinyue Yu, Mengxuan Zou, Francesca Spiga, Sarah Dawson, George Davey Smith, Julian P T Higgins

**Affiliations:** Population Health Sciences, Bristol Medical School, University of Bristol, Bristol, United Kingdom; Cancer Research UK Integrative Cancer Epidemiology Programme, University of Bristol, Bristol, United Kingdom; Nutrition Group, Great Ormond Street Institute of Child Health, University College London, London, United Kingdom; Population Health Sciences, Bristol Medical School, University of Bristol, Bristol, United Kingdom; Population Health Sciences, Bristol Medical School, University of Bristol, Bristol, United Kingdom; Population Health Sciences, Bristol Medical School, University of Bristol, Bristol, United Kingdom; Population Health Sciences, Bristol Medical School, University of Bristol, Bristol, United Kingdom; Population Health Sciences, Bristol Medical School, University of Bristol, Bristol, United Kingdom; Cancer Research UK Integrative Cancer Epidemiology Programme, University of Bristol, Bristol, United Kingdom

**Keywords:** Mendelian randomization, risk of bias, quality assessment, systematic review, evaluation tools

## Abstract

**Background:**

The growing use of Mendelian randomization (MR) has heightened the need for rigorous quality and bias assessment tools. A previous systematic review included studies published up to July 2021 identified 14 structured instruments for conducting, evaluating, and reporting MR studies. However, methodological developments have accelerated in the years since.

**Methods:**

We updated the previous systematic review to include tools published between July 2021 and January 2025, applying the same search strategy and eligibility criteria. Two reviewers independently screened articles, extracted data, and mapped tool content to bias domains.

**Results:**

We identified 15 additional articles, bringing the total to 29 tools. Of these 29, 19 provided structured evaluation tools. Twelve of the 19 evaluation tools were newly added in the present review, which addressed broader methodological domains beyond core instrumental variable assumptions, including genetic instrument selection, population stratification, sensitivity analyses, and dataset considerations. However, substantial variation in bias domains, structure, and scoring methods across tools persists. Key gaps remain in the assessment of linkage disequilibrium, missing data, and dynastic effects.

**Conclusions:**

While the number of structured tools has increased in recent years, the lack of standardization across tools makes it difficult to compare across systematic reviews of MR studies. Developing more complete and standardized evaluation frameworks and properly testing these tools in practice are important next steps to improve the overall quality of MR research.

Key Messages

- We updated a systematic review of tools for assessing the conduct, evaluation, or reporting of Mendelian randomization analyses.
- Twenty-nine tools were found, with newer tools covering a broader range of methodological domains, including explicit assessment of core instrumental variable assumptions such as genetic instrument selection (relevance) and population stratification (independence).
- There are large differences between tools in structure, scoring methods, and bias domains covered, and some important areas such as linkage disequilibrium and missing data remain under-assessed.

## Introduction

Mendelian randomization (MR) has become a highly used approach in epidemiological research, offering a robust method for causal inference, generally through leveraging genetic variants as instrumental variables (IVs) [1–4]. The appeal of MR lies in its ability to reduce biases from confounding and reverse causation, common limitations in conventional observational studies, making it a valuable tool for strengthening causal inference [3, 5].

With the rapid expansion of MR applications across various domains of biomedical research, there has been an accompanying growth in concern regarding the robustness, reproducibility, and interpretability of many published MR studies. Editorials and commentaries have raised alarm over the uncritical or superficial use of MR methods, prompting calls for more rigorous and standardized approaches to evaluating study quality and potential biases [4, 6–8]. The complexity of MR studies, including the selection of appropriate genetic instruments, assumptions underpinning IV analysis, and sensitivity to population structure, necessitates structured tools to guide researchers in evaluating methodological robustness [8, 9]. Over the years, several tools have been developed to aid in the appraisal of MR studies, providing structured frameworks for evaluating risk of bias, assessing validity, and ensuring methodological consistency [10]. Despite these efforts, no universally accepted, standardized approach to MR risk of bias and quality assessment has been established. Variability in assessment criteria and inconsistency in tool usage across studies highlight the need for continued refinement and harmonization of evaluation methods. Addressing this gap is essential to enhance the credibility and reproducibility of MR findings, particularly in the context of systematic reviews and meta-analyses.

A previous systematic review identified and evaluated tools for assessing risk of bias and study quality in MR research published up until the first half of 2021 [10]. The review by Spiga *et al.* identified 14 tools designed for the evaluation, conduct, and/or reporting of MR studies, with seven tools specifically developed for risk of bias or quality assessment. These tools addressed key MR-specific biases, particularly those related to IV assumptions, genetic instrument selection, and population/sample characteristics. While these three core assumptions underpin valid causal inference, unbiased point estimation additionally requires homogeneity or monotonicity assumptions, such as linearity and absence of effect modification, which some newer tools have begun to evaluate. Building on the earlier review, further investigation is warranted to identify additional tools that have emerged since 2021 following the publication of STROBE-MR [8] and to evaluate the value of these tools in supporting the conduct, evaluation, and reporting of MR studies in systematic reviews. We therefore updated the systematic review to identify newly developed tools, or tools adapted from existing frameworks, for assessing MR studies. Our goals are to facilitate selection of a suitable existing tool by authors of systematic reviews of MR studies and to inform development of a new tool to assess risk of bias in MR following the successful Risk Of Bias in Non-randomized Studies (ROBINS) framework [11].

## Materials and methods

### Eligibility criteria

We retained the eligibility criteria used by Spiga *et al.* [10] (PROSPERO CRD42021282836) though restricted our update to newly developed or significantly modified structured tools for evaluating MR conduct, evaluation, and/or reporting, maintaining consistency with Spiga *et al.*’s “review of tools”. Articles were included if they: (i) present structured tools—such as guidelines, checklists, or frameworks—designed or proposed to support comprehensive assessment of the design, conduct, and/or reporting of MR studies, or to provide systematic guidance through the process of conducting or reporting such studies; and (ii) be available as full-text articles, including peer-reviewed publications, preprints, or published protocols published since July 2021. We excluded articles that merely referenced other tools included in the review (e.g. STROBE-MR) without substantial modification or that did not employ a structured evaluation method.

### Search strategy

We systematically searched MEDLINE (Ovid), Embase (Ovid), Web of Science, preprint servers (bioRxiv and medRxiv), PROSPERO, and Google Scholar for articles published since Spiga *et al.*’s search in July 2021 up to 24 January 2025. We adopted the same search strategy as Spiga *et al.* for MR tools (see Supplementary Material, Section S1). Moreover, to identify further tools, we followed up a convenience sample of the 45 systematic review protocols listed in Spiga *et al.* [10], examining the subsequent reviews to identify any additional eligible tools (or tool modifications) that had been used. No language restrictions were imposed. References from identified articles were screened for additional eligible articles.

### Study selection and data extraction

Two reviewers (J.Y. and M.Z.) independently screened titles and abstracts for relevance using Rayyan (www.rayyan.ai; see Supplementary Material, Section S2). Full texts of potentially eligible articles were independently assessed by the same reviewers, with discrepancies resolved through discussion or by consulting a third reviewer (F.S.). For each included study, we extracted details about: the type of tool (newly developed or modified); specific IV assumptions assessed; criteria used for assessment and any scoring system applied; and practical application of the tool in MR research or systematic reviews.

### Data synthesis

We summarized findings in a narrative synthesis, describing the characteristics of new or modified MR risk of bias tools. Tool classification was based on their stated purpose. Where tools spanned multiple categories, this was reflected in Table 1. Additional explanation is provided in Supplementary Material, Section S3. To ensure consistency and comparability with the original review by Spiga *et al.* [10], we adopted their methodological approach for categorizing assessment items within each identified tool according to the specific type of bias or methodological concern they addressed, and subsequently grouped these into clearly defined bias-related domains.

**Table 1:** Details of included studies containing one or more tools for evaluating, conducting, and reporting MR studies.

Tools	Study ID	Type of report	Type of tool	Scope of the tool(s)^a^	Number of tools	Structure of the tool
**1**	**Boef 2015 [27]**	Systematic review of MR studies	Checklist for reporting MR studies	Reporting	1	Checklist
**2 & 3**	**Burgess 2020 [19]**	Tool proposal	Guideline for performing MR investigations	Assessing	2	1. Domain-based checklist (assessing and reporting) 2. Flowchart (conducting)
				Conducting		
				Reporting		
**4**	**Davey Smith 2019 [33]**	Tool proposal	STROBE-MR; guideline for the reporting of MR studies	Reporting	1	Checklist
**5**	**Davies 2018 [34]**	Tool proposal	Guide, checklist and glossary for MR studies	Assessing	1	Checklist
				Reporting		
**6**	**Grau-Perez 2019 [22]**	Systematic review of MR studies	Adapted the criteria from Longnecker 1988 [35] for observational studies and Boef 2015 [27] for MR studies to assess study quality	Assessing	1	Domain-based evaluation chart
				Reporting		
**7**	**Grover 2017 [30]**	Tool proposal	Guideline for performing MR analysis	Conducting	1	Flowchart
**8**	**Kuźma 2018 [36]**	Systematic review of MR studies	Q-Genie tool for assessing quality of genetic association studies [37]	Assessing	1	Rating scale
				Reporting		
**9**	**Lawlor 2019 [31]**	Tool proposal	MR dictionary and online tool	Conducting	1	Flowchart
**10**	**Lee 2020 [38]**	Systematic review of MR studies	Structured checklist	Assessing	1	Checklist
				Reporting		
**11**	**Lor 2019 [28]**	Systematic review of MR studies	Proposed checklist and guideline for reporting	Reporting	1	Checklist
**12**	**Mamluk 2020 [23]**	Systematic review of MR studies	Self-developed tool for MR quality/risk of bias	Assessing	1	Bias domain-based rating
**13**	**Swerdlow 2016 [32]**	Tool proposal	Conceptual guidance/checklist principles	Conducting	1	Decision tree
**14**	**Treur 2021 [26]**	Systematic review of MR studies	Self-developed scoring system	Assessing	1	Scoring system
				Reporting		
Updates 2021–2025						
**15**	**Alhassan 2024 [14]**	Systematic review of MR studies	Assumption-based assessment	Assessing	1	Assumption-based rating
**16**	**Bouajila 2024 [18]**	Systematic review of MR studies	Q-Genie tool plus self-developed evaluation of IV assumptions	Assessing	1	Domain based rating
				Reporting		
**17**	**Burgess 2023 [7]**	Tool proposal	Updated checklist and guidance from Burgess 2020 [19]	Assessing	1	Checklist
**18**	**Gao 2024 [20]**	Systematic review of MR studies	Scored system among three domains	Assessing	1	Domain-based scoring system
**19**	**Gibson 2023 [21]**	Meta-epidemiological study of MR studies	Checklist, adapted from STROBE-MR 2021 [9] and Burgess 2020 guidelines [19]	Assessing	1	25-item checklist
				Reporting		
**20**	**Lee 2024 [12]**	Systematic review of MR studies	A quality rating adapted from Mamluk 2020 [23]	Assessing	1	Bias domain-based rating
**21**	**Luo 2024 [15]**	Review of MR studies	Adapted quality framework from Boef 2015 [27]	Assessing	1	Assumption-based rating
**22**	**Luo 2022 [16]**	Systematic review of MR studies	Structured risk of bias framework	Assessing	1	Assumption-based rating
**23**	**Markozannes 2022 [13]**	Systematic review of MR studies	Self-developed framework for assessing robustness of the evidence in MR studies	Consistency of the evidence	1	Grading flowchart/framework
**24**	**Nguyen 2024 [39]**	Tool proposal	Structured tool for MR study evaluation	Assessing	1	Checklist
				Reporting	1	
**25**	**Omar 2024 [17]**	Systematic review of MR studies	Adapted from Luo 2022 [16]	Assessing	1	Assumption-based rating
**26**	**Rosas-Chavez 2025 [25]**	Systematic review of MR studies	Quantitative scoring system across domains	Assessing	1	Scoring system
**27**	**Skrivankova 2021 [8]**	Tool proposal	Updated from *STROBE 2007 [40] reporting guideline*	Reporting	1	Checklist
**28**	**Visontay 2022 [24]**	Systematic review of MR studies	Risk of bias tool adapted from Mamluk 2020 [23]	Assessing	1	Bias domain-based rating
**29**	**Woolf 2022 [29]**	Tool proposal	Checklist of reporting quality	Reporting	1	Checklist

a Scope of the tools: Reporting, conducting, assessing (assessing risk of bias/quality of evidence), and robustness of the evidence. “Checklists” present yes/no or descriptive items; rating scales assign graded levels of concern; scoring systems provide numeric summaries; evaluation charts combine multiple bias domains in matrix form; domain-based ratings group items by bias domain (e.g. instrument strength, pleiotropy).

Abbreviations: IV = instrumental variable; MR = Mendelian randomization; STROBE = Strengthening the Reporting of Observational Studies in Epidemiology; 2SMR = two-sample Mendelian randomization.

In line with the original review, we anticipated that most tools would fall into one of three previously established categories: (i) tools designed to support the conduct of MR studies; (ii) tools to assess risk of bias or study quality; and (iii) tools to guide reporting. Where any newly identified tools did not fit these categories, we classified them under a new category, if appropriate, to reflect their distinct purpose.

As this review synthesizes methodological tools rather than intervention or outcome data, a formal assessment of quality, certainty, or reporting bias was not applicable.

## Results

### Summary of the screening

**Figure 1:**
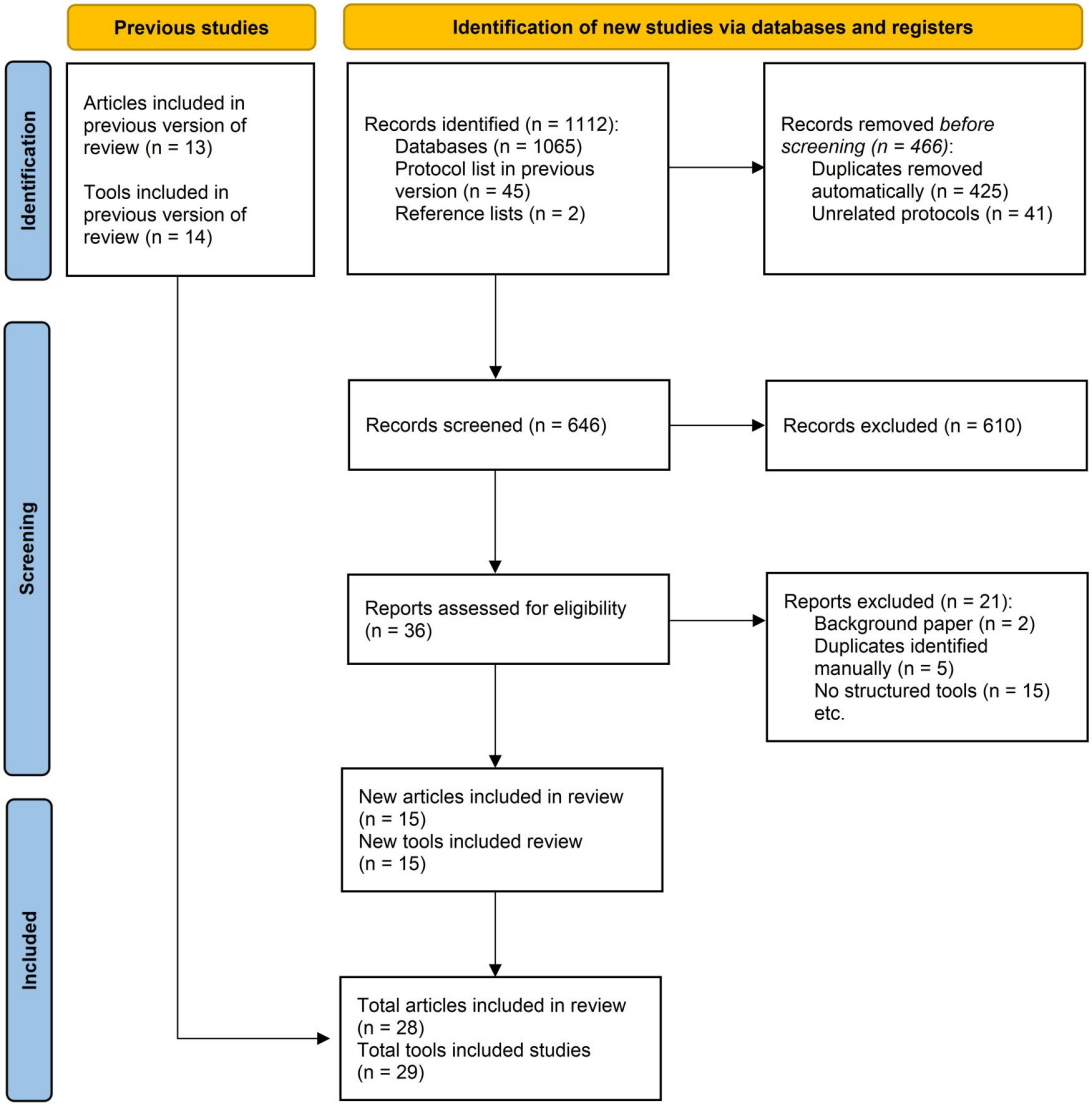
Flow diagram illustrating the identification, screening, and inclusion of articles containing tools for the conduct, evaluation, and reporting of Mendelian randomization studies.

Our updated literature search identified 1112 records (Fig. 1), plus seven potentially relevant articles from the 45 protocols listed in Spiga *et al.* [10]. After deduplication, 646 records underwent title and abstract screening, with 36 full-text articles assessed for eligibility. Excluded articles are listed in Supplementary Material, Section S4. Fifteen new tools met our inclusion criteria, including two identified from protocols [12, 13]. Combined with the 14 tools from Spiga *et al.* [10], 29 tools were included in this updated review.

Table 1 summarizes the scope of these tools. Nineteen were structured tools specifically designed for assessing methodological quality or risk of bias in MR studies (seven from Spiga’s review, 12 newly identified). These tools included assessments of IV assumptions [14–17], domain-based checklists or rating scales [18–24] and novel scoring metrics [25, 26]. Thirteen tools included assessment of reporting quality; five explicitly focused on reporting quality [8, 21, 27–29]; and eight addressed both reporting and evaluation within one tool. Four tools provided structured guidance for conducting MR studies [19, 30–32] and one introduced a new category focused on assessing “robustness of the evidence” [13].

Burgess *et al.* [19], uniquely addressed all three purposes of evaluating, conducting, and reporting in two separate tools in 2020, and then substantially revised and published the guidelines in 2023 [7]. The updated version retains the dual structure (checklist and flowchart) but has expanded its content and scope. It now includes 10 thematic sections covering motivation, data sources, variant selection, harmonization, primary and sensitivity analyses (including robust methods), data presentation, and interpretation. This revision also introduces new guidance on within-family MR to address biases such as dynastic effects and assortative mating, offers updated recommendations for drug–target MR, and provides a detailed summary table of robust MR methods.

### Tools for evaluating quality/risk of bias of the MR study

Table 2 summarizes the bias-related domains addressed by the 19 tools. In total, 150 items addressed biases/quality related issues, 77 of which directly related to the three core IV assumptions. All tools explicitly addressed the exclusion restriction (IV3), emphasizing consistent attention towards horizontal pleiotropy. Most addressed the independence assumption (IV2, 84%), particularly in relation to confounding and the relevance assumption (IV1, 89%), with a focus on weak instrument bias. Frequently evaluated additional domains included sensitivity analyses (74%), sample overlap (42%), and population heterogeneity (42%). Less commonly addressed domains included choice of controls (5%) which applies only to MR studies using case-control or disease outcome designs, construction of genetic score (5%) and missing data (11%).

**Table 2:** Details of specific Mendelian randomization bias and limitation addressed by items or questions within each assessing tool.^a^

Bias/topic domain	Specific bias or topic addressed by tool	Burgess 2020	Davies 2018	Grau-Perez 2019	Kuźma 2018	Lee 2020	Mamluk 2020	Treur 2021	Gao 2024	Gibson 2023	Alhassan 2022	Visontay 2022	Rosas-Chavez 2025	Luo 2022	Luo 2024	Bouajila 2024	Burgess 2023	Omar 2024	Nguyen 2024	Lee 2024	Total items
**IV1-relevance**	Choice of variants	Y							Y	Y			Y			Y	Y		Y		6
	Weak instrument bias		Y	Y	Y	Y	Y	Y	Y	Y	Y	Y	Y	Y	Y	Y		Y	Y	Y	17
**IV2-independence**	Choice of variants	Y							Y	Y			Y				Y		Y		6
	Confounding		Y	Y	Y	Y	Y	Y			Y	Y	Y	Y	Y	Y	Y	Y	Y	Y	16
	Population stratification						Y		Y	Y			Y	Y	Y				Y	Y	7
**IV3-exclusion restriction**	Choice of variants	Y							Y	Y			Y				Y		Y		6
	Horizontal pleiotropy	Y	Y	Y	Y	Y	Y	Y	Y	Y	Y	Y	Y	Y	Y	Y	Y	Y	Y	Y	19
**Genetic instrument**	Choice of variants	Y							Y	Y			Y			Y	Y		Y		6
	Construction of genetic score							Y													1
	Variants harmonization	Y	Y			Y		Y	Y	Y			Y								7
	Linkage disequilibrium	Y	Y			Y							Y	Y							4
**Population/sample**	Samples overlap	Y	Y			Y		Y		Y			Y		Y					Y	8
	Population heterogeneity	Y	Y	Y		Y	Y	Y	Y					Y						Y	8
	Choice of controls			Y																	1
	Selection bias			Y								Y			Y						3
**Sensitivity analysis**	Evidence of robustness	Y	Y			Y		Y	Y	Y	Y		Y	Y	Y	Y	Y	Y	Y	Y	14
**Measurement error**	Exposure measurement error/misclassification			Y	Y			Y	Y	Y				Y		Y		Y			7
	Outcome measurement error/misclassification			Y	Y			Y	Y	Y						Y					5
**Missing data**	Missing data			Y						Y											2
**Other confounding**	Non-MR-specific confounding			Y	Y		Y									Y				Y	4
**Other sources of bias**	Traditional epidemiologic bias (i.e. non-MR-specific)				Y^b^								Y^c^			Y^d^					3

a This table summarizes whether each of the 19 methodological quality and risk of bias tools explicitly assessed a given domain. A tick (Y) indicates that the domain was clearly and explicitly included in the tool’s assessment criteria. Domains assessed by most tools reflect widespread consensus on their importance. Conversely, domains addressed by only a few tools may reflect either under-recognition, context-specific relevance, or gaps in existing tools. The interpretation should consider the evolving nature of MR methodology and the intended purpose of each tool.

b Bias related to ““study justification” bias”.

c Bias related to “choice of genome-wide association study (GWAS) dataset” or dataset currency (Statistical power?).

d Bias related to “assessment of non-linearity”.

Abbreviations: IV = instrumental variable; MR = Mendelian randomization; Y = yes.

Additionally, three tools assessed additional sources of bias not covered by standard domains: Kuźma *et al.* [33] addressed bias related to “study justification”; Rosas-Chavez and Merriman [25] included statistical power and genome-wide association study (GWAS) dataset choice; and Bouajila *et al.* [18] evaluated non-linearity.

### Tools for addressing conducting and reporting quality of MR study

Among the 13 reporting tools, the number of domains ranged from 3 to 6, with all tools addressing reporting of the three core IV assumptions. Woolf *et al.* [29] included items addressing homogeneity and sample overlap (specifically in two-sample MR studies), while Gibson *et al.* [21] addressed linkage disequilibrium and heteroscedasticity. For tools guiding conduct of MR studies, Grover *et al.* [30], Lawlor *et al.* [31], and Swerdlow *et al.* [32] covered between 5 and 10 domains, including items addressing the selection of genetic instruments, assumptions validation, handling of pleiotropy, and statistical analyses.

### A new tool addressing consistency of the evidence from an MR study

We identified a newly developed framework that focuses on evaluating the strength and consistency of MR findings: Markozannes *et al.* [13] proposed a structured decision flow that classifies each exposure–outcome association into one of four levels, which they call “robust evidence”, “probably”, “suggestive”, and “insufficient”. Their classification depends on whether the main MR estimate reaches statistical significance and whether results from additional methods, such as MR-Egger, weighted median, MR-PRESSO, or multivariable MR, show consistent direction and significance. We view this as an assessment of consistency rather than robustness; indeed others have reconceived the options as “consistent”, “concordant”, “inconsistent”, and “inadequate” [34]. We created a new category of tool to reflect this focus on consistency within a study.

### Other aspects for consideration of MR study

We summarize details of 57 items addressing any other aspects of the MR analysis in Table 3. Among these, we found two items in two tools addressing clinical implications of MR results [35, 36]; six items in six tools addressing the datasets used [7, 21, 25, 35–37]; 35 items in 17 tools addressing the genetic instrument, including genetic variant selection rationale [7, 15–21, 24, 25, 36], method used to obtain variants [7, 14–18, 20–22, 24, 25, 36], and variant strength [13–18, 20, 21, 24–26, 36]; 23 items in 10 tools addressing the interpretation of MR analysis results, including applicability, transportability, temporality, and bidirectional effects [7, 15, 18, 20, 21, 24–26, 35, 36]; 12 items in 12 tools addressing the MR rationale [7, 15–19, 21, 24–26, 33, 36]; 19 items in 15 tools addressing the MR results [7, 13–18, 20–22, 24, 25, 35–37]; five items in five tools addressing precision of the results, including statistical power or sample size [22, 25, 26, 33, 36]; eight items in five tools addressing the selection of the population(s) or sample(s) [13, 15, 19, 21, 25]; 36 items in 12 tools addressing statistical analysis methods and any related details including reporting of primary and secondary analyses [7, 13–15, 19–22, 25, 26, 33, 36]; and five item in five tools addressed the type of dataset used [7, 19, 21, 25, 36].

**Table 3:** Details of other MR-relevant content of items or questions within each assessing tool.

Aspect of MR analysis	Specific topic	Burgess 2020	Davies 2018	Grau-Perez 2019	Kuźma 2018	Lee 2020	Mamluk 2020	Treur 2021	Gao 2024	Gibson 2022	Alhassan 2022	Visontay 2022	Rosas-Chavez 2025	Luo S 2022	Luo 2024	Bouajila 2024	Burgess 2023	Omar 2024	Nguyen 2024	Lee 2024
**Clinical implications**	Reporting of clinical implication		Y																Y	
**Datasets**	Reporting of datasets used		Y			Y				Y			Y				Y		Y	
**Genetic instrument**	Reporting of MR rationale (biological rationale)	Y							Y	Y		Y	Y	Y	Y	Y	Y	Y	Y	
	Reporting of method used to obtain genetic variant			Y					Y	Y	Y	Y	Y	Y	Y	Y	Y	Y	Y	
	Reporting of genetic variant strength							Y	Y	Y	Y	Y	Y	Y	Y	Y		Y	Y	Y
**Interpretation**	Applicability/transportability		Y						Y				Y		Y					
	Interpretation of results		Y						Y	Y		Y	Y		Y		Y		Y	
	Reporting of MR estimates for interpretation of results		Y							Y			Y						Y	
	Bidirectional effects							Y	Y				Y							
	Temporality							Y												
**MR rationale**	Reporting of MR rationale	Y			Y			Y		Y		Y	Y	Y	Y	Y	Y	Y	Y	
**MR results**	Reporting of comparison of MR estimate with observational study estimate		Y	Y		Y											Y		Y	
	Reporting of MR analysis results	Y	Y	Y					Y	Y	Y	Y	Y	Y	Y	Y	Y	Y	Y	Y
**Precision**	Statistical power/sample size			Y	Y			Y					Y			Y			Y	
**Selection of population/sample**	Gathering specific methods details	Y								Y			Y		Y					Y
	Reporting of relevance of the selected population to the research question	Y													Y					Y
**Statistical analysis**	Reporting of statistical methods			Y	Y				Y	Y	Y		Y			Y			Y	Y
	Reporting of primary analysis statistical methods	Y								Y			Y						Y	Y
	Reporting of primary analysis details	Y						Y		Y			Y						Y	Y
	Reporting of secondary analysis statistical methods	Y							Y	Y	Y		Y		Y				Y	Y
**Type of dataset**	Gathering specific methods details	Y								Y			Y				Y		Y	

Abbreviations: MR = Mendelian randomization; Y = yes.

## Discussion

In this updated systematic review, we identified 12 new structured tools for assessing quality and risk of bias in MR studies, doubling the number in Spiga *et al.* [10]. Of the 29 tools included, 19 focused mainly on evaluating MR quality and bias; this reflects increasing recognition of the need for systematic assessment of MR study quality. Newer tools retain strong coverage of the three core IV assumptions while broadening scope to include genetic instrument selection, population stratification, sensitivity analyses, and other methodological aspects. However, linkage disequilibrium, missing data, and dynastic effects remain under-addressed. Notably, only Treur *et al.* [26] explicitly addressed exposures that vary over time, despite temporality being a critical determinant of causal interpretation in MR studies.

We identified a new category of tool focused on evaluating consistency of evidence (termed “robustness” by the authors). The framework by Markozannes *et al.* [13] classifies MR associations into levels of evidence based on consistency across multiple analytical methods. Unlike most tools, it aims to support interpretation of results by classifying each MR association into levels of evidence, depending on agreement across various robustness checks. This may complement risk of bias tools by indicating whether a causal interpretation is supported consistently across analyses, though consistency does not guarantee validity, as shared biases may persist.

Another development is the 2023 update of the Burgess *et al.* guidelines [7], which extends their earlier dual-tool framework for evaluation and conduct into a more comprehensive and methodologically detailed framework. Key additions include new sections on within-family MR, updated recommendations for drug–target analyses and variant selection, and expanded coverage of robust statistical methods and triangulation strategies. Visual tools, such as flowcharts, structured reviewer checklist, and a comparative table of MR methods, further enhance its practical utility. These enhancements reflect ongoing concerns about methodological rigour and a shift towards more formalized quality and bias assessment.

Despite broader bias coverage, challenges remain. One key issue is the variation in evaluation approaches. Tools differ in wording and different structures (e.g. checklists, ratings, and scoring systems), making it difficult to compare results. To assist researchers in selecting suitable tools, we summarized key characteristics such as tool structure, intended use, and domain coverage. While we are reluctant to recommend a specific tool, our preference is for domain-based tools and checklists, which evaluate the potential for bias in specific areas of study conduct [38], such as Burgess *et al.* [7] and Mamluk *et al.* [23]. In practice, tools with operationalized criteria and structured guidance may suit systematic reviewers, whereas brief checklists may be more practical for applied researchers conducting primary analyses. Numerical scoring systems pose challenges due to arbitrary item weighting, which can be difficult to justify [39]. Another key issue is distinguishing domains relevant only in specific MR contexts from those broadly applicable yet under-assessed. Sample overlap, homogeneity and bidirectional effects mainly apply to two-sample or multivariable MR and may be underrepresented for this reason. In contrast, linkage disequilibrium, missing data, and dynastic effects are methodologically relevant across many MR settings yet remain inconsistently covered. These represent clear gaps for future tool development.

Moreover, a fundamental but often underappreciated assumption in MR is gene–environment equivalence [40, 41], which requires that genetic proxies for exposures exert effects that mimic those of modifying the exposures through feasible means. Closely linked to the consistency assumption [42], this issue is increasingly recognized as central to MR validity and has already been noted in STROBE-MR [8, 9] and the previous Spiga review [10]. In this review, only a few tools mention it [7, 12, 15, 36], with Nguyen *et al.* [36] treating it as an independent checklist item. This may be because the assumption is challenging to assess since it is inherently context‐specific, difficult to operationalize for a standardized checklist, and often cannot be judged objectively for all exposures. We suggest that while gene–environment equivalence should be mentioned in frameworks of MR quality assessment, its evaluation may best be framed as a qualitative plausibility check rather than a scored item applicable universally [42].

We also identified a reduced emphasis on reporting-specific tools following the publication of STROBE-MR in 2021, which has since become the accepted reporting standard. However, the included reporting tools still add value by operationalizing or extending STROBE-MR. For instance, Woolf *et al.* [29] developed a detailed two-sample MR-specific reporting checklist that included items on data harmonization and instrument construction, and Gibson *et al.* [21] introduced a practical checklist for applied researchers.

The strengths of this updated review include its timely response to the rapid expansion of methodological research in MR, doubling the number of evaluation tools compared with the previous review. We ensured consistency with the original search strategies and inclusion criteria, strengthening reliability. We also highlighted additional bias sources not yet categorized, such as study justification [33], and assessment of non-linearity [18], which may inform future frameworks. By consolidating recent developments, we hope to accelerate progress towards a robust and standardized tool for assessing bias in MR studies, supporting their appropriate use within triangulated evidence approaches in epidemiology [4, 6, 43].

We also acknowledge limitations. Our classification of items by bias type was subjective and others may reach different conclusions. We excluded guidelines and tools for general IV analyses, focussing instead on comprehensive MR-specific frameworks expected to accommodate extensions beyond the classical IV structure. While we searched key sources of grey literature, we did not include conference proceedings, theses, or institutional repositories and did not inspect a large sample of systematic reviews. Therefore, some tools may have been missed.

As highlighted in our review and recent publications [4, 6, 44], concerns persist regarding the quality and interpretability of many MR studies; these reinforce the need for structured tools and wider adoption to improve transparency, reproducibility, and methodological rigour. While we focused on MR-specific tools for consistency with Spiga *et al.*, future work may benefit from incorporating relevant frameworks from the broader IV literature. We also acknowledge that the boundaries between tools intended for conducting, reporting and evaluating MR studies are not always clear-cut. In practice, many tools serve overlapping functions and may be repurposed across different research contexts. This observation highlights the value of future efforts to integrate and harmonize guidance across these domains. Further research should also develop standardized and semi-automated approaches to improve consistency, while reducing the burden on systematic reviewers and editors.

## Supplementary Material

dyag052_Supplementary_Data

## Data Availability

All data are presented in tables and Supplementary Materials.

## Use of artificial intelligence (AI) tools

The authors confirm that all conceptualization, analysis, data synthesis, and interpretation were conducted by the authors. ChatGPT (OpenAI, GPT-5) was used solely to assist with language editing of the article text. The authors reviewed and approved all AI-assisted edits to ensure accuracy and integrity. We used Rayyan solely as a screening platform for collaborative title/abstract and full-text screening by two independent reviewers. Deduplication was performed using the in-built function in databases prior to import into Rayyan. We did not use Rayyan’s AI-assisted prediction feature during screening to ensure that all inclusion decisions were based solely on human judgement.
